# A comparison of three types of web-based inhibition training for the reduction of alcohol consumption in problem drinkers: study protocol

**DOI:** 10.1186/1471-2458-14-796

**Published:** 2014-08-05

**Authors:** Andrew Jones, Elly McGrath, Katrijn Houben, Chantal Nederkoorn, Eric Robinson, Matt Field

**Affiliations:** Department of Psychological Sciences, University of Liverpool, United Kingdom and UK Centre for Tobacco and Alcohol Studies (UKCTAS), Liverpool, L69 7ZA UK; Clinical Psychological Science, Maastricht University, Maastrich, The Netherlands

**Keywords:** Alcohol, Disinhibition, Inhibitory control, Inhibition training

## Abstract

**Background:**

Problem drinkers have poor inhibitory control (disinhibition). Previous studies have demonstrated that various forms of ‘inhibition training’ can reduce alcohol consumption in the laboratory and at short-term follow-up, but their longer-term efficacy and mechanisms of action are unknown. In this phase 2 randomised controlled trial we will contrast the effects of three forms of inhibition training and a control intervention, delivered via the Internet in multiple sessions over four weeks, on alcohol consumption in heavy drinkers.

**Methods/design:**

Heavy drinkers who are interested in reducing their alcohol consumption will receive a brief intervention and will monitor their own alcohol intake for one week before being randomised to one of four treatment groups: 1. General inhibition training; 2. Cue-Specific inhibition training; 3. Alcohol No-Go training; or 4. Control. They will complete up to 14 sessions of training via the Internet over a four-week period, and will be followed-up for a further six weeks after the end of the training period. Primary outcome measures are reductions in alcohol consumption and heavy drinking days. The number of abstinent days is a secondary outcome measure. We will also investigate changes in inhibitory control and automatic alcohol affective associations in response to training.

**Discussion:**

This study will establish if web-based inhibition training can help problem drinkers to reduce their alcohol intake, and it will identify which form(s) of inhibition training are most effective.

**Trial registation:**

Trial Registation number: ISRCTN55671858.

## Background

Disinhibition - the inability to suppress, delay or change a response that is no longer required or is inappropriate - is a core feature of both impulsivity and executive functioning [1, 2]. The construct can be measured using computerised tasks such as the Stop-Signal (SST [3]) and Go/No-Go tasks (GNGT [4]). In these tasks, participants must override a dominant motor response when faced with a ‘Stop’ or ‘No-Go’ cue, respectively. Disinhibition is associated with individual differences in drug and alcohol consumption [5, 6], it predicts hazardous drinking [7] and distinguishes alcoholics from healthy controls [8]. Disinhibition is not merely a consequence of problem drinking because it *precedes* changes in alcohol involvement: it predicts future drinking behaviour in adolescents [9], slow development of inhibitory processes during adolescence predicts problem drinking later in life [10] and disinhibition predicts the likelihood that heavy drinkers will transition to alcohol dependence [11].

Disinhibition is not a stable trait, but rather a transient state that fluctuates in response to environmental triggers. Alcohol-related cues provoke temporary increases in disinhibition in heavy drinkers [12, 13] and alcoholics [14, 15]. Other arousing cues, acute stress, and self-control depletion also lead to increased disinhibition, and it is hypothesised that these fluctuations in disinhibition increase the risk of drinking to excess [1, 16]. Encouragingly, inhibitory control can be improved with practice. One study [17] demonstrated that repeatedly practicing the SST led to improved inhibitory control, whereas in another [18] key parameters of the SST were changed such that it became more difficult over time. Both studies demonstrated progressive improvements in inhibitory control that were accompanied by changes in markers of brain activity that are associated with inhibitory control (see also [19, 20]).

Inhibitory control is malleable and it has a causal influence on problem drinking, which makes it a viable target for clinical interventions [16, 21]. We demonstrated that priming restrained behaviour during the SST led to improvements in inhibitory control and reductions in alcohol consumption in the laboratory [22, 23]; comparable effects have been reported on food intake [24, 25] and gambling [26, 27]. Another laboratory study demonstrated that if participants completed a modified SST in which they learned to inhibit behaviour only in the presence of alcohol-related cues, this led to an improvement in inhibitory control that was specific to alcohol cues, and was accompanied by a reduction in alcohol consumption in the laboratory [28]. Thus, training of both *cue*-*specific* and *general* inhibitory control may prompt reductions in alcohol consumption. Other studies demonstrated that pairing inhibition with alcohol-related cues during a modified GNGT did not directly improve disinhibition (measured with the SST), but it did change affective associations with alcohol and led to reduced alcohol consumption after a one week follow-up [29, 30]; see also [31]).

These recent laboratory studies suggest that training of inhibitory control using modified versions of the SST and GNGT can lead to reductions in alcohol consumption, although the underlying mechanisms may differ across tasks. We suggest that this is because of distinct inhibitory and associative learning processes that are engaged during the GNGT and the SST. The GNGT measures automatic or bottom-up inhibition because the decision to inhibit is made as soon as the No-Go cue is detected, and this inhibition can be consistently paired with a particular stimulus such as an alcohol-related cue [32, 33]. On the other hand, the SST measures controlled or top-down inhibition because the stop signal is not presented until after a motor response has been initiated, which makes it more difficult to pair inhibition with a particular stimulus such as an alcohol cue. Given these differences between tasks, training inhibitory control using the SST may not generalise to improved performance on the GNGT, and vice versa [29, 33]. Furthermore, automatic inhibition leads to devaluation of No-Go cues [34], whereas there is no evidence that controlled inhibition alters the valuation of Stop signals.

Laboratory studies involving a single session of inhibition training are an important first step in establishing proof of concept that such training could be a viable clinical intervention. However, research into other types of cognitive training for alcohol problems suggests that multiple sessions of training are needed to produce longer-lasting reductions in alcohol consumption and cognition that generalise outside of the training context [35–38]. Related to this, a recent study on inhibition training for weight loss demonstrated that repeated food-related GNGT training via the internet led to significant weight loss that was maintained over four weeks [39]. Other interventions for problem drinking are also effective if delivered online in multiple sessions [40]. Overall, it appears that i) interventions targeting behaviour change are more effective if repeated in multiple sessions and ii) delivery of such interventions via the Internet can be efficient, cost-effective and efficacious [41, 42].

### Aims and hypotheses

The aim of this study is to examine the efficacy of repeated inhibition training as an intervention to reduce alcohol consumption in heavy drinkers recruited from the local community. We will recruit individuals who consume alcohol in excess of UK government guidelines for safe drinking, and who are motivated to cut down [43]. Most heavy drinkers never seek treatment [44, 45], and without treatment they are likely to transition into more harmful patterns of drinking [46]. Heavy drinkers who are not severely alcohol dependent are also less likely to engage with abstinence based treatments [47]. However, a significant proportion of regular drinkers express desires to reduce their alcohol consumption [48]. Therefore a reduction in alcohol consumption is a viable goal for interventions for this population [49, 50].

In order to ensure that they are motivated to reduce their drinking, all participants will complete a brief intervention (‘Down Your Drink’ (DYD) [51]) followed by one week of monitoring of their alcohol consumption before they begin inhibition training. This brief intervention and self-monitoring period are also included in the study for pragmatic purposes. Many heavy drinkers will reduce their alcohol consumption after a brief intervention and/or a period of self-monitoring, and failure to control for these ‘non-specific’ effects can obscure effects of active treatments [52, 53].

Participants will be randomly allocated to one of four groups: 1. General inhibition training; 2. Cue-Specific inhibition training; 3. Alcohol No-Go training; or 4. Control. All participants will complete an internet intervention 14 times over a four-week period (every other day, on average). Their alcohol consumption will be monitored throughout the training period. We predict reductions in alcohol consumption during the training period in the first three groups compared to the control group. This will be the first study to directly compare these three types of inhibition training, so we have no *a priori* hypotheses about which will produce the largest reduction in alcohol consumption.

We will monitor participants’ alcohol consumption at two, four and six-weeks after the end of the intervention (follow-up). We predict that any group differences in alcohol consumption that are seen at the end of the training period will be maintained throughout the follow-up period.

We will also investigate psychological changes that occur as a result of inhibition training. We predict that Alcohol No-Go training will cause changes in automatic alcohol affective associations [29, 34], whereas General inhibition training will lead to non-specific improvements in inhibitory control [18], and Cue-Specific inhibition training will lead to improvements in inhibitory control that are specific to alcohol cues [28].

## Methods/design

### Participants

Participants will be recruited from the local community through advertisements in the local media and via employers and local community organisations. Eligible participants will i) be aged between 25–65, ii) drink over UK government guidelines for safe drinking ([43]: more than 14 UK units for females and 21 UK units for males, where 1 UK unit = 8 grams of alcohol)) iii), be interested in trying to reduce their alcohol consumption.

Exclusion criteria include i) any history of treatment for an alcohol use disorder, and ii) a current or previous diagnosis of substance use disorder (including alcohol use disorder), and/or attention deficit disorder, because these conditions are associated with poor inhibitory control [54]. Participants will be required to have access to the Internet from a desktop or laptop computer, and an email address. The study received ethical approval from the Institute for Psychology, Health and Society Research Ethics Committee.

#### Sample size and randomisation

We used G*Power 3.1.5 [55] to calculate the required sample size for power of 0.80 to detect any main effect or interaction (numerator df = 3) at an alpha level of 0.05 (see Table 1). We based this calculation on a recent meta-analysis concerning e-help interventions for problem drinkers [41] which demonstrated a medium effect size (f = 0.25). As seen in Table 1 we require a total sample size of 179 to detect an effect.

**Table 1:** Sample size calculation using G*Power3

Analysis:	A priori: compute required sample size		
Input:	Effect size f	=	0.25
	α err prob	=	0.05
	Power (1-β err prob)	=	0.8
	Numerator df	=	3
	Number of groups	=	4
Output:	Noncentrality parameter λ	=	11.1875000
	Critical F	=	2.6562339
	Denominator df	=	175
	**Total sample size**	**=**	**179**
	Actual power	**=**	0.8015073

A recent study with problem drinkers who had a goal of controlled drinking (rather than abstinence) demonstrated that 33% of study participants reduced their alcohol intake in response to a period of self-monitoring before randomisation to experimental treatments [52]. As argued in that study, it may be important to exclude these ‘early reducers’ from primary analyses because a floor effect may render their drinking unresponsive to experimental treatments. Therefore, we increased our sample size to 268 (N = 67 per experimental group) so that we are powered to exclude up to 33% of the final sample from analyses if they report a large reduction in alcohol consumption before randomisation, if necessary. In line with the internet and ecological momentary assessment research which informed our methods, we expect attrition to be approximately 10% [56, 57].

Initial group allocation will be double-blind: A random number generator will be used to randomly assign participants to experimental groups *after* the initial one-week period [58]. Researchers will therefore be aware of group allocation during the training phase, but they will have no direct contact with the participants aside from generic e-mails and reminders containing the links to inhibition training tasks for each day.

## Materials

### Questionnaires

During their initial visit to the laboratory, participants will complete the two-week timeline follow-back alcohol consumption diary (TLFB [59]), the Alcohol Use Disorders Identification Test (AUDIT [60]), the Barratt Impulsivity Scales version 11 (BIS [61]) and the Temptation and Restraint Inventory (TRI [62]).

#### Pictorial stimuli

Ten alcohol-related and 10 matched neutral pictures, a subset of those used in our previous inhibition training study [28], will be used in the training and control tasks. Alcohol-related pictures depict alcoholic drinks that are common in the UK, and individuals holding or consuming those drinks, whereas neutral images depict pictures of stationery and office furniture. Picture pairs are matched on perceptual characteristics such as complexity and brightness. Smaller subsets of these picture pairs are used in the Cue-Specific SST and the Alcohol Valence Implicit Association Task (see below).

#### Computer tasks

All tasks will be programmed and administered using Inquisit 3.0 [63]. Baseline and follow-up tasks will be completed on a laptop or desktop PC in a lab at the University of Liverpool, whereas online tasks will be hosted by Inquisit 4.0 web edition [64].

#### Tasks administered in the laboratory

*Standard SST* [65]: We will use a standard version of the task that incorporates arbitrary visual stimuli (the letters X and O) along with the tracking procedure to set the stop signal delay (SSD). The initial delay will be 250 ms and will increase by 50 ms after every successful inhibition and decrease by 50 ms after every failure to inhibit. The critical variable is Stop Signal Reaction Time (SSRT), the estimate of stopping latency.

*Cue*-*Specific SST* [66]: Participants will categorise images of alcoholic drinks based on their orientation (portrait or landscape). The same dynamic algorithm and number of trials as the standard task will be used. A measure of alcohol cue-specific SSRT will be computed from this task. Eight alcohol pictures will be used in the task.

*Alcohol Valence Implicit Association Task* (AVIAT) [29]: Participants will be required to categorise stimuli into two target categories (Alcohol or Neutral images) and two affective attribute categories (Positive and Negative words). The *D* measure ([67] will be calculated as an indicator of the strength of alcohol-positive associations. Six picture pairs will be used in the task.

#### Training tasks administered via internet

Both Cue-Specific and General SST training will be implemented using modified versions of the SST. The Alcohol No-Go training takes the form of a modified GNGT. The Control intervention is similar to the Cue-Specific SST with the crucial difference that it contains no Stop signals. Each training session will take approximately seven minutes to complete.

**Figure 1:**
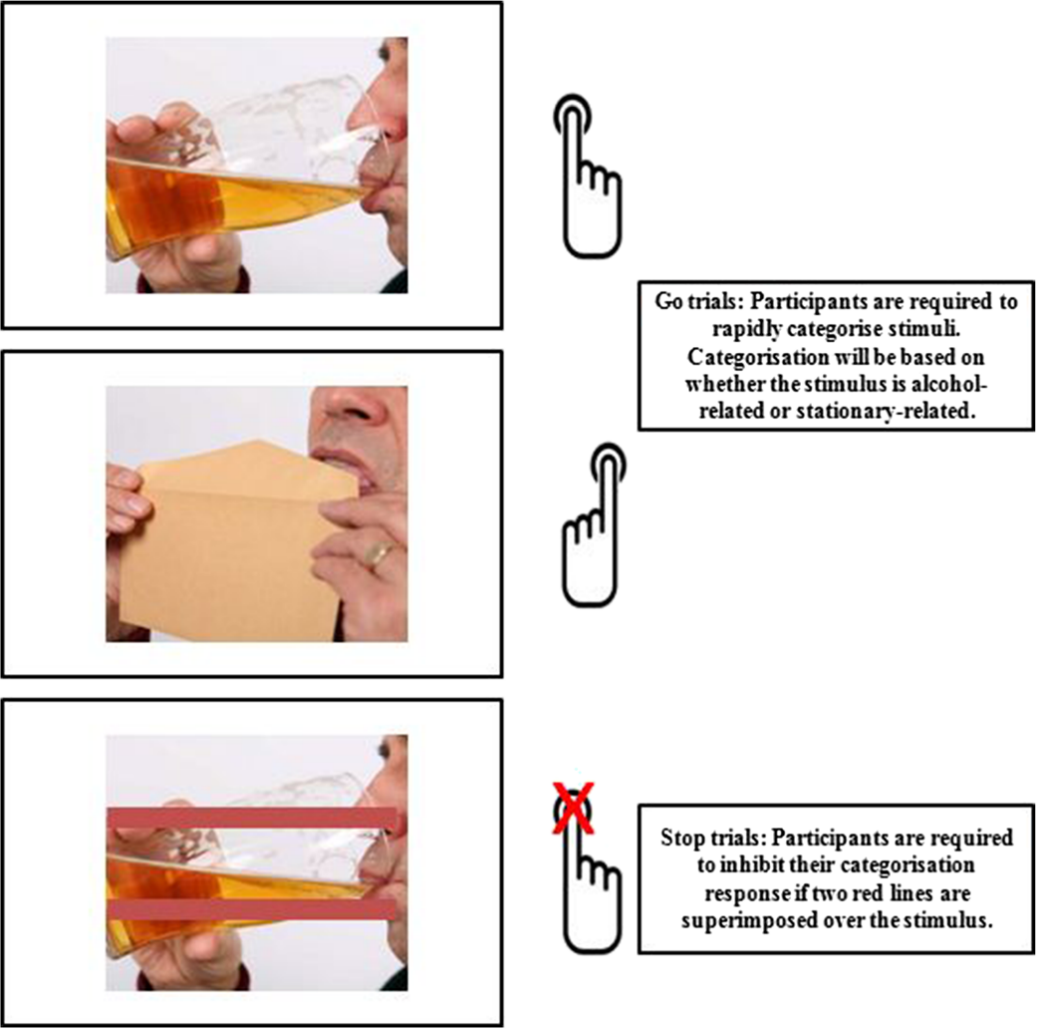
**Schematic representation of trials in Cue-Specific inhibition training.**

*Cue*-*Specific inhibition training* (*see Figure *1): Participants will be required to rapidly categorise pictures as alcohol-related or stationery-related, by pressing the ‘X’ or the ‘M’ key on their keyboard. There will be 200 trials separated by a short break after half the trials. On 75% of all trials in each block this categorisation will not be interrupted; these are Go trials. On the remaining 25% of trials, two red lines (the Stop Signal) will be superimposed over the picture after a fixed SSD. Participants will be instructed to inhibit their response to the picture whenever they see the Stop signal, and wait for the next trial. The Stop Signal will appear on 50% of alcohol trials, but never on neutral trials. Therefore, participants should learn the contingency that presentation of an alcohol related picture may signal inhibition, but this is not always the case. This is important because cue-specific inhibition training requires participants to learn the contingency between alcohol and inhibition, but we cannot present the stop-signal on 100% of alcohol trials (50% of total trials), because this would encourage participants to adopt a waiting strategy, particularly in the presence of alcohol cues [68].

Training will be adaptive based on performance over sessions. It will become progressively more difficult as performance improves, thereby leading to improved cue-specific inhibition over time. The initial fixed SSD will be 250 ms and in the first session each stop trial will implement this delay. Once participants are able to inhibit to at least 50% of stop trials during a block at this delay, the SSD in the following block will increase by 10 ms in the next session, making inhibition more difficult. Therefore, the maximum (most difficult) SSD at the end of training will be 380 ms [250 ms + (10 ms X 13 sessions with potential increments)].

**Figure 2:**
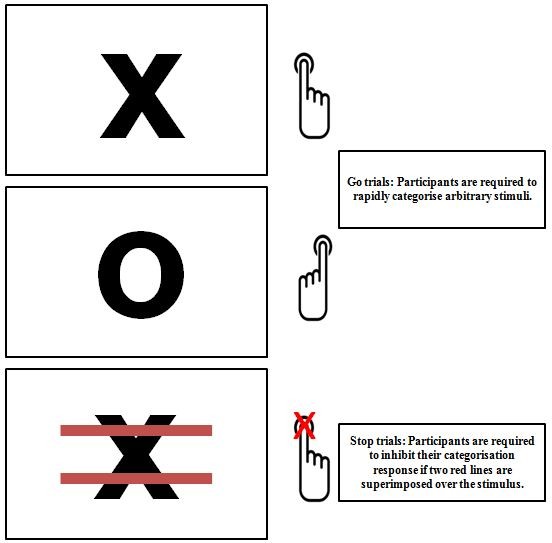
**Schematic representation of trials in General inhibition training.**

*General Inhibition training* (*see Figure*2): This group will complete a modified version of the Stop-Signal task using arbitrary stimuli (the letters X and O). In this version the Stop Signal will be superimposed over 50% of the X stimuli and none of the O stimuli. There will be 200 trials in total, 25% of which are Stop Signal trials. As with Cue-Specific inhibition training, the initial SSD will be set at 250 ms and if participants successfully inhibit on at least 50% of Stop trials within a block the SSD will increase by 10 ms in the next block.

**Figure 3:**
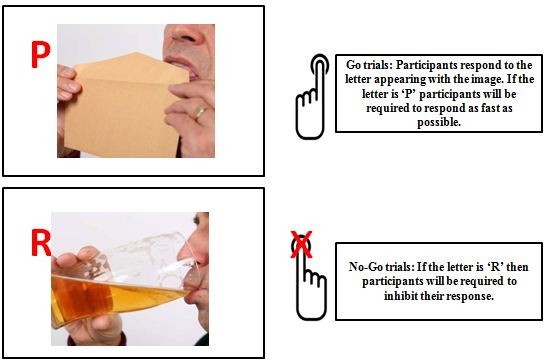
**Schematic representation of trials in Alcohol No-Go training.**

*Alcohol No*-*Go training* (*based on* 29; *see Figure*3): Participants in this group will respond to a letter that will be presented in the corner of an alcohol-related or neutral picture. The letter P is the ‘Go’ cue which signals that participants should respond, whereas the letter R is the ‘No-Go’ cue which signals that they should not respond on that trial. The Go cue will always appear in the corner of neutral pictures and the No-Go cue will always appear in the corner of alcohol pictures. Each block contains 200 trials: 100 Go and 100 No-Go.

*No training* (*control*) *group*: Participants will perform 200 trials of a forced choice reaction time task in which they rapidly categorise alcohol-related and neutral pictures by pressing the keys ‘X’ and ‘M’. They will never be required to inhibit their responses.

### Procedure

**Figure 4:**
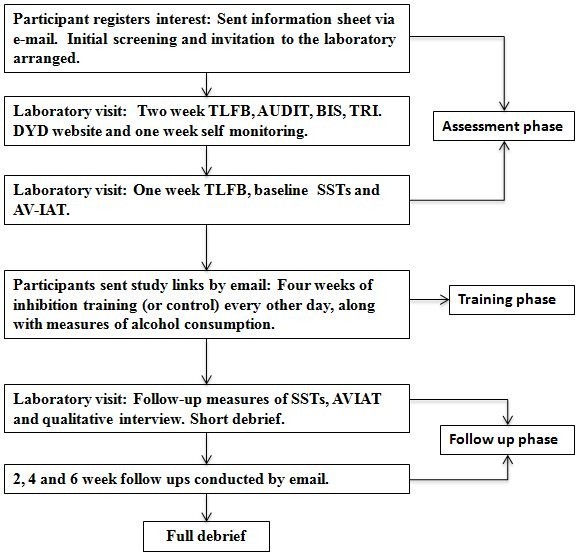
**Flow chart of trial procedure.**

Figure 4 shows a flow chart of the flow procedure.

#### Assessment phase

Participants who register interest in the study will complete a brief email screen to assess eligibility before attending an initial appointment. They will provide informed consent, before completing a questionnaire battery consisting of the TLFB, AUDIT, BIS and TRI. Participants will then be asked to set up their own account on the DYD website. DYD is an interactive web-based intervention for people who want to cut down on drinking [51]. Participants will complete the ‘quick visit’ option on the site. This involves participants making a decision about whether they need to cut back on their drinking and if so, they make a goal to cut back on drinking for the next week. They will also be asked to use an online drinking diary on the site to keep a record of their alcohol consumption over the next week. Participants will then leave the laboratory and return one week later. Participants who do not wish to reduce their drinking will be able to withdraw at this point.

Upon their return participants will check their online drinking diary and use this to complete a paper-and-pencil TLFB for the previous week. Participants will then be asked to re-affirm consent that they wish to continue in the study; if they wish to withdraw they will receive compensation (£20) before release. Participants who choose to remain in the study will complete baseline measures of the general SST, cue-specific SST and the AV-IAT in the laboratory. Participants will be told that they should continue to use the DYD drinking diary for the remainder of the study, and they should complete training tasks by following Internet links that will be emailed to them.

#### Training phase

Participants will be randomised to one of four experimental groups: (1) Cue-Specific inhibition training, (2) General inhibition training, (3) Alcohol No-Go training or (4) Control (no training). Participants will complete up to 14 web-based training sessions during the four weeks of the training phase by following links that will be emailed to them every other day. In order to increase compliance, participants will be informed that if they miss a session on a particular day they can complete the session on the following day, but they must complete at least two sessions per week and there must not be a gap of more than three days between sessions. If they have not completed a session before the next one is due, a reminder email will be sent. They will be informed they can access the training at any point between midday to midnight.

Each time they log on to the site they will be asked how much alcohol they have consumed since their previous session (in UK units). A new email will be sent out containing the link for each assessment. Participants will also be provided with an email address and phone number in case they encounter any problems, and they will be contacted after two weeks by email to ensure that they are not experiencing problems. If participants have completed fewer than half of the required sessions at this stage (<4) they will be withdrawn from the study. These measures are in place to ensure high rates of compliance [69]. After the four weeks of training sessions, participants will return to the laboratory and complete follow up questionnaires including the AUDIT, TLFB and a measure of how motivated they were to reduce their alcohol consumption during the previous four weeks. They will also complete the SSTs (cue-specific and standard) and AVIAT in the laboratory once more. They will be reimbursed for their participation up until that point (£40-£130, depending on the number of training sessions completed) and partially debriefed. Participants are not told which group they were allocated to at this point.

#### Follow up phase

Participants will be sent a link via email two, four and six weeks after their final assessments. Each link will direct them to a two-week TLFB hosted on Inquisit web. Participants will be offered £5 for each follow-up session completed. Following the final follow-up, all participants (regardless of whether they completed the follow-up assessments) will be sent a full debrief.

A small, random subsample of participants (10) from each active treatment group will be invited back to the University following the final debrief for a semi-structured interview with a researcher about their experiences during the intervention. This interview will include open-ended questions in order to probe their views regarding the accessibility of the training program and any potential barriers to use. We will use qualitative analysis to identify common themes relating to user accessibility and experience.

#### Outcome measures

The pre-defined co-primary outcome measures are (1) the number of units of alcohol consumed, and (2) the number of heavy drinking days, defined as alcohol consumption ≥ 60 g (7.5 UK units) for men and ≥ 40 g (5 UK units) for women [70] on any given day. The number of abstinent days is our secondary outcome measure.

#### Primary analyses

Primary and secondary outcome measures will be analysed using mixed-design ANOVA with a between-subject factor of treatment condition (4: Cue-Specific inhibition training, General inhibition training, Alcohol No-Go training, Control) and a within-subject factor of time (3: baseline, two weeks into training, four weeks into training).

#### Supplementary analyses

If there are group differences in outcome measures at the end of training, we will analyse follow-up data using a separate mixed-design ANOVA with a between-subject factor of treatment condition, and a within-subject factor of time (3: two-week, four-week and six-week follow-up).

The effects of treatment condition on general inhibition, cue-specific inhibition and automatic alcohol-affective associations will be analysed using mixed-design ANOVA with a between-subject factor of treatment condition, and a within-subject factor of time (2: before training vs. after training).

The responses from the subgroup of participants who return to the laboratory for an interview about their experiences of taking part in the trial will be analysed using thematic analyses to identify themes related to treatment efficacy, acceptability and barriers to use (see [71]).

Any further exploratory analyses will be labelled as exploratory in the final publication.

## Discussion

This study protocol describes the design of a randomised controlled trial to determine the efficacy of three different types of inhibition training delivered via the Internet for the reduction of alcohol consumption in heavy drinkers. To our knowledge, this will be the first study to examine i) the effects of repeated inhibition training on alcohol consumption and ii) compare between three different forms of inhibition training: Cue-Specific, General, and Alcohol No-Go training. This study also builds on theoretical models and recent laboratory research by investigating the psychological changes that occur as a result of inhibition training, namely changes in automatic alcohol affective associations, cue-specific inhibitory control and general inhibitory control.

A strength of the intervention is its delivery via the Internet, which means that most participants will be able to complete it at home. This overcomes problems of limited availability and accessibility which afflict other interventions for problem drinking [72]. This is particularly important given that most heavy drinkers are unable or unwilling to access conventional treatments [73]. A limitation of any web-based study is high attrition [42]. However, we will adopt methods used in similar studies such as structured incentives, repeated visits to the lab and regular reminder emails, to mitigate this risk [56, 69]. Also, by administering computerised tasks outside of the laboratory environment a certain amount of control is lost, and participants may be susceptible to distractions in their home environment. This limitation may be offset by the observation that interventions which are administered in the environment in which alcohol consumption usually occurs are more likely to be effective than similar interventions administered in clinical settings [74, 75].

## Abbreviations

AUDIT: Alcohol Use Disorders Identification Test
AVIAT: Alcohol Valence Implicit Association Task
BIS: Barratt Impulsivity Scale
DYD: Down Your Drink
GNGT: Go/ No-Go task
SST: Stop-Signal Task
SSD: Stop Signal Delay
SSRT: Stop Signal Reaction Time
TLFB: Timeline Follow-Back.
